# White matter microstructure in youth with and at risk for bipolar disorder

**DOI:** 10.1111/bdi.12885

**Published:** 2020-01-21

**Authors:** Julia O. Linke, Caitlin Stavish, Nancy E. Adleman, Joelle Sarlls, Kenneth E. Towbin, Ellen Leibenluft, Melissa A. Brotman

**Affiliations:** ^1^ Emotion and Development Branch National Institute of Mental Health National Institutes of Health Bethesda MD USA; ^2^ Department of Psychology The Catholic University of America Washington DC USA; ^3^ NIH MRI Research Facility National Institute of Neurological Disorders and Stroke National Institutes of Health Bethesda MD USA

**Keywords:** age, anxiety, bipolar disorder, DTI, emotion regulation, first‐degree relatives, irritability, TBSS

## Abstract

**Objectives:**

Bipolar disorder (BD) and familial risk for BD have been associated with aberrant white matter (WM) microstructure in the corpus callosum and fronto‐limbic pathways. These abnormalities might constitute trait or state marker and have been suggested to result from aberrant maturation and to relate to difficulties in emotion regulation.

**Methods:**

To determine whether WM alterations represent a trait, disease or resilience marker, we compared youth at risk for BD (n = 36 first‐degree relatives, REL) to youth with BD (n = 36) and healthy volunteers (n = 36, HV) using diffusion tensor imaging.

**Results:**

Individuals with BD and REL did not differ from each other in WM microstructure and, compared to HV, showed similar aberrations in the superior corona radiata (SCR)/corticospinal tract (CST) and the body of the corpus callosum. WM microstructure of the anterior CC showed reduced age‐related in‐creases in BD compared to REL and HV. Further, individuals with BD and REL showed in‐creased difficulties in emotion regulation, which were associated with the microstructure of the anterior thalamic radiation.

**Discussion:**

Alterations in the SCR/CST and the body of the corpus callosum appear to represent a trait marker of BD, whereas changes in other WM tracts seem to be a disease state marker. Our findings also support the role of aberrant developmental trajectories of WM microstructure in the risk architecture of BD, although longitudinal studies are needed to confirm this association. Finally, our findings show the relevance of WM microstructure for difficulties in emotion regulation—a core characteristic of BD.

## 1 INTRODUCTION

Bipolar disorder (BD), which is characterized by discrete, episodic changes in affect, motivation, cognition and behavior,^1^ is a severe mental disorder with a prevalence of at least 1%^2^ and a high heritability of 60%‐80%.^3^ To date, the pathophysiology of BD is still poorly understood impeding early and precise diagnosis,^4^ and thereby detrimentally affecting the course of the disease.^5^ Studies in individuals with BD indicate that the disorder relates to disturbances in fronto‐limbic networks relevant for emotion processing and regulation.^6^ However, to differentiate quantitative risk factors from markers of disease progression, it is necessary to also study healthy individuals at high risk to develop bipolar disorder (ie, unaffected first‐degree relatives of individuals with BD; REL). In addition, studies in individuals at risk for BD before the first peak of onset that occurs between 21‐25 years^7^) offer the possibility to identify adaptive functional changes associated with resilience and will thus further advance our etiological understanding of BD.

The aberrant development of white matter (WM) pathways interconnecting the prefrontal cortex with limbic brain regions, which previously showed aberrant activity in individuals with and at risk for BD,^8^ has been argued to be central to the emergence of BD.^9^ The microstructure of WM pathways can be quantified using diffusion tensor imaging (DTI) and is often described in terms of fractional anisotropy (FA), which is positively correlated with increased directional coherence, increased axon packing density, and smaller axon diameter in WM.^10^ In adult BD, reduced FA in the anterior corpus callosum (CC) and the cingulum bundle has emerged as the most robust finding in several meta‐analyses.^11^, ^12^ In addition, reduced FA in association fibers (eg, superior longitudinal fasciculus [SLF], anterior thalamic radiation [ATR]) and projection fibers (eg, uncinate fasciculus [UNC], corticospinal tract [CST]) has been frequently reported in adult BD (for review see Ref. 13). Of note, 12 studies were conducted in pediatric BD and, as in adult BD, reduced FA in the anterior CC, cingulum bundle, UNC, SLF, CST and ATR,^14^ emerged as the most robust findings. Moreover, seven studies in unaffected adult REL reported reduced FA in the CC,^15^ cingulum bundle,^16^ SLF,^16^, ^17^ ATR,^15^, ^17^ and UNC.^15^, ^16^, ^17^ Based on these findings, it has been argued that altered WM microstructure might represent a quantitative risk marker for BD, meaning it plays an antecedent, possibly causal, role in the pathophysiology of BD.^13^ However, the two studies that compared adolescent REL (n = 25‐79, age: 15‐21) to HV's yielded conflicting results. While one study reported reduced FA in the CC, CST, UNC, IFOF, ILF, SLF, and ATR,^15^ the other found FA increases in the CC, UNC, ILF, SLF, and cingulum bundle.^18^ Thus, more DTI studies in youth at risk are needed to discern whether these abnormalities truly reflect the risk for BD.

In this context it is also of interest that little is known as to how and when WM abnormalities in BD develop. It has been proposed that failed pruning of axons and dendrites between early childhood and puberty, particularly in prefrontal‐limbic tracts such as UNC, impair top‐down emotion regulation in BD.^9^ Others have proposed that alterations in the normal developmental myelination of fiber tracts, which continues until the third decade of life^19^ or altered myelin plasticity in response to experiences^20^ contribute to the risk for BD.^13^ However, studies addressing these developmental hypotheses are scarce. A cross‐sectional study implied age‐related FA increases in the CC, cingulum bundle and UNC in HV, but FA decreases in BD.^21^ Consistent with this observation, the absence of normative FA increases in the UNC was observed in a longitudinal study using a region of interest (ROI) approach that followed individuals with BD (n = 27) for 2.5 years during adolescence/young adulthood.^22^ However, a 2‐year longitudinal study could not identify differential developmental trajectories of WM in REL (n = 69) compared to HV during adolescence/young adulthood.^23^ Despite these interesting findings, more studies are needed to advance our understanding how WM alterations in BD develop.

Finally, it has been hypothesized that abnormal WM microstructure contribute to emotion regulation difficulties in BD.^9^ However, no studies have directly tested the link between WM microstructure and difficulties in emotion regulation, although there is some evidence that WM microstructure particularly in the anterior CC relates to symptoms of negative affect indicative of disturbed emotion regulation.^24^

The present study is the first to directly compare WM microstructure in an adolescent sample of 36 individuals diagnosed with BD, 36 HV, and 36 REL to discern risk from disease and resilience markers. Based on the literature, we expected widespread FA reductions in BD and possibly less pronounced FA alterations in overlapping areas in REL. Second, we explored group*age interactions in significant tracts and the CC and UNC that were previously associated with differential developmental trajectories in BD.^21^, ^22^ Compared to HV, we expected reduced age‐related FA increases in BD but not in REL. Third, we sought to determine the contribution of WM microstructure to the difficulties in different dimensions of emotion regulation, which we assessed with the Difficulties in Emotion Regulation Scale^25^ (DERS).

## 2 METHODS

### 2.1 Participants and diagnostic assessment

We recruited 108 youths (aged 8‐21 years) diagnosed with BD (n = 36: n = 15 BD 1, n = 21 BD 2), related to an individual with BD (n = 36: n = 12 siblings, n = 21 offspring, n = 3 sibling + offspring) and HVs with no family history of BD (n = 36). Study groups were comparable with regard to sex, IQ and socioeconomic status,^26^ but differed in age (BD > REL: *P* = .001; BD > HV: *P* = .042; REL vs HV: *P* = .38). Most of the BD subsample was euthymic; four participants met criteria for a hypomanic episode, and one met criteria for a mixed episode. BD and REL were comparable regarding depressive symptoms (*P* = .13), levels of irritability (*P* = .08) and anxiety (*P* = .10). BD youth showed more manic symptoms (*P* < .001) and lower psychological and social functioning (*P* < .001) than REL. The number of BD youth and REL with an anxiety disorder was comparable. ADHD was more frequently diagnosed in BD youth (*P* = .01). Five individuals with BD and 31 REL were unmedicated (see Table 1).

**Table 1:** Demographics and clinical characterization of the study sample

	BD patients (n = 36)	Relatives (n = 36)	Controls (n = 36)	Statistics^a^	*P*‐value
Sex (female/male)	17/19	17/19	20/16	χ^2^_(2)_ = 0.67	.71
	Mean (SD)	Mean (SD)	Mean (SD)		
Age, y	16.8 (3.27)	14.0 (3.46)	15.0 (2.81)	*F*_(2,105)_ = 7.30	.001
Intelligence score, mean (SD)	109.4 (11.42)	112.3 (11.04)	109.6 (12.02)	*F*_(2,105)_ = 0.71	.50
SES Hollingshead^b^, mean (SD)	51.8 (31.89)	51.2 (33.03)	40.6 (17.46)	*F*_(2,88)_ = 1.38	.26
Medication load, mean (SD)	3.2 (2.69)	0.1 (0.35)	0.0 (0.0)	*t*_(70)_ = 6.75	<.001
Pubertal stage					
Breast/genital	4.2 (1.25)	3.3 (1.45)	3.7 (1.28)	H_(2)_ = 7.84	.02
Pubic hair	4.1 (1.23)	3.6 (1.55)	3.7 (1.40)	H_(2)_ = 2.38	.30
Current symptoms					
CGAS	53.4 (12.44)	81.9 (12.45)	N/A	*t*_(70)_ = 9.58	<0.001
YMRS	5.9 (5.68)	1.9 (2.84)	N/A	*t*_(70)_ = 3.83	<0.001
CDRS^b^	24.1 (9.80)	21.2 (4.93)	N/A	*t*_(66)_ = 1.55	.13
ARI^b^	3.5 (3.36)	2.2 (2.64)	0.7 (1.24)	*F*_(2,104)_ = 10.42	<0.001
SCARED^b^	9.7 (10.41)	14.5 (11.56)	5.4 (5.40)	*F*_(2,102)_ = 7.68	.001
	No. (%)	No. (%)	No. (%)		
Current KSADS diagnoses					
ADHD	18 (50)	8 (22)	N/A	χ^2^_(1)_ = 6.02	.01
Anxiety disorders	16 (44)	10 (28)	N/A	χ^2^_(1)_ = 2.16	.14
GAD	12 (33)	2 (6)	N/A	χ^2^_(1)_ = 8.87	.003
SAD	4 (11)	5 (14)	N/A	χ^2^_(1)_ = 0.13	.72
SoPhob	7 (19)	5 (14)	N/A	χ^2^_(1)_ = 0.40	.53
	0/1/2/≥3	0/1/2/≥3	0/1/2/≥3		
Psychotropic medication					
Total	5/7/7/17	31/5/0/0	N/A	χ^2^_(1)_ = 43.11	<0.001
Antidepressants	28/7/0/1	36/0/0/0	N/A	χ^2^_(1)_ = 9.00	.011
Antiepileptics	22/12/2/0	36/0/0/0	N/A	χ^2^_(1)_ = 17.38	<.001
Lithium	24/12/0/0	36/0/0/0	N/A	χ^2^_(1)_ = 14.40	<.001
Antipsychotics	17/16/2/0	36/0/0/0	N/A	χ^2^_(1)_ = 25.81	<.001
Tranquilizer	29/5/2/0	36/0/0/0	N/A	χ^2^_(1)_ = 7.75	<.001
Stimulants	17/17/2/0	31/5/0/0	N/A	χ^2^_(1)_ = 12.62	.002

Abbreviations: ADHD, attention deficit/hyperactivity disorder; BD, bipolar disorder; CD, conduct disorder; CDRS, Children's Depression Rating Scale; DMDD, disruptive mood dysregulation disorder; GAD, generalized anxiety disorder; HV, healthy volunteers; n, sample size; ODD, oppositional defiant disorder; SAD, separation anxiety disorder; SD, standard deviation; SoPh, social phobia; y, years; YMRS, Young Mania Rating Scale.

a Different degrees of freedom reflect missing data.

b Data unavailable: ARI: 1 REL; CDRS: 3 BD, 1 REL; SCARED: 2 BD, 1 HV; SES Hollingshead: 4 BD, 4 REL, 9 HV.

Participants over age 18 and parents of minor participants gave written informed consent after receiving a complete description of the study; minors gave written assent. Procedures were approved by the Institutional Review Board of the National Institute of Mental Health.

### 2.2 Clinical assessment

All participants were assessed with the Kiddie Schedule for Affective Disorders and Schizophrenia (K‐SADS) with the DMDD supplement^27^ by master's and doctoral‐level clinicians. A senior clinical psychologist (MAB) or psychiatrist (EL, KT) confirmed the primary diagnosis. Exclusion criteria comprised neurological disorders, autism spectrum disorders, substance use within the last two months, conditions for which MRI is contraindicated, and full‐scale IQ <70, measured using the Wechsler Abbreviated Scale of Intelligence (WASI).^28^

Manic and depressive symptoms were assessed by master's and doctoral‐level clinicians, with excellent inter‐rater reliability (κ >0.9 for all ratings) within a week of scanning in BD youth and REL using the Young Mania Rating Scale (YMRS)^29^ and the Children's Depression Rating Scale (CDRS)^30^; respectively. Irritability and anxiety symptoms were rated by both children and parents with the Affective Reactivity Index (ARI)^31^ and the Screen for Child Anxiety Related Disorders (SCARED).^32^ Global functioning was assessed in BD youth and REL by master's and doctoral‐level clinicians using the Children's Global Assessment Scale (CGAS).^33^

Child and parent completed the DERS,^34^ which has shown good internal reliability and convergent validity in adolescents.^35^ The DERS assess 6 dimensions of emotion regulation: (1) awareness and understanding of emotional responses, (2) acceptance of emotions, (3) the ability to control impulsive behaviors when experiencing negative emotions, (4) the ability to employ situationally appropriate emotion regulation strategies to meet one's goals, (5) the ability to engage in goal‐directed behavior while experiencing negative emotions, and (6) the extent to which one is clear about the emotions one is experiencing. The total scale and subscales were scored to indicate the frequency with which difficulties in emotion regulation are experienced, with scores ranging from 1 (“Almost never”) to 5 (“Almost always”). The mean scores from parent and child ratings are presented in Table 2.

**Table 2:** Scores on the Difficulties in Emotion Regulation Scale

	BD patients		Relatives		Controls		Statistics	*P*‐value	*P*‐values of post‐hoc ANCOVAs		
	Mean	SD	Mean	SD	Mean	SD			BD vs REL	BD vs HV	REL vs HV
Total score	95.8	23.16	72.0	19.30	52.4	9.91	*F*_(2,104)_ = 44.1	<.001	<.001	<.001	<.001
Emotional awareness	15.9	4.07	14.9	3.29	12.4	3.85	*F*_(2,104)_ = 6.6	.002	.084	.001	.020
Non‐Acceptance of negative emotions	12.9	3.13	9.3	2.96	7.7	1.72	*F*_(2,104)_ = 23.1	<.001	<.001	<.001	.033
Impulse control	18.1	4.70	11.1	4.64	8.0	3.1	*F*_(2,104)_ = 36.2	<.001	<.001	<.001	.012
Access to emotion regulation strategies	20.9	4.68	13.8	4.94	10.1	3.60	*F*_(2,104)_ = 34.6	<.001	<.001	<.001	.005
Goal‐directed behavior	17.4	5.09	13.7	4.89	8.7	2.42	*F*_(2,104)_ = 32.0	<.001	<.001	<.001	<.001
Emotional clarity	11.0	2.57	9.6	2.79	8.2	2.37	*F*_(2,104)_ = 7.1	.001	.026	<.001	.055

### 2.3 DTI data acquisition

MRI data were acquired on a General Electric 3.0 Tesla scanner with a 32‐channel head coil. DTI was performed using a single‐shot echo‐planar imaging sequence with robust fat suppression (TR = 6751 ms, TE = 79 ms, 62 axial slices, 2.5 mm slice thickness, FoV = 240 mm^2^, matrix size = 96 × 96) acquiring five images without diffusion weighting and 75 images with diffusion gradients (6: b = 300 s/mm^2^, 69: b = 1000 s/mm^2^) for each slice distributed across four runs. In case of excessive motion, a 5th run containing the diffusion directions of the previously compromised images was acquired to reduce the likelihood that results are caused by differential subject motion. Here, we define excessive motion as the percentage of outliers per diffusion‐weighted volume (number of outlier voxels for each diffusion‐weighted volume divided by the total number of brain voxels) being larger than the empirically determined threshold of 1.5%.^36^ The number of reacquired volumes ranged between 0‐18 (0%‐14%) in all diagnostic groups. Further, T2‐weighted data obtained from a fast spin‐echo sequence with fat suppression (TR = 7500 ms, TE = 100 ms, 100 axial slices, slice thickness = 1.7 mm, no gap, FoV = 240 × 192 mm, matrix size = 256 × 192) were used for preprocessing. The acquisition of the DTI data could last up to 20 minutes, depending on the number of reacquired volumes, plus an additional 2.5 minutes for the T2‐weighted image. All MRI data were visually inspected to exclude anatomical abnormalities.

### 2.4 Processing of DTI data

Before processing, all diffusion data were visually inspected by NEA and JOL. Corrupted volumes showing common DTI artifacts such as ghosting or geometric distortions^37^ were removed. Volumes with excessive motion were replaced by the reacquired volumes unless the reacquired volumes showed motion artifacts themselves. Although not all bad volumes could be replaced (range of included volumes = 74‐81 in all three diagnostic groups), there were no group differences in the number of volumes after preprocessing (*F*_(2,105)_ = 1.04, *P* = .26). Next, the T2‐weighted image was reoriented into a common space defined by the mid‐sagittal plane, the anterior commissure, and the posterior commissure, using mipav.

DTI data were pre‐processed using the software package TORTOISE version 1.4.0.^38^ First, diffusion‐weighted images were corrected for motion and eddy current distortion. There were no significant group differences in the six rigid body motion parameters (Tx: *F*_(2,105)_ = 0.04, *P* = .97; Ty: *F*_(2,105)_ = 1.20, *P* = .31; Tz: *F*_(2,105)_ = 0.28, *P* = .76; Rx: *F*_(2,105)_ = 0.04, *P* = .97; Ry: *F*_(2,105)_ = 0.07, *P* = .93; Rz: *F*_(2,105)_ = 1.33, *P* = .27, see Figure S1). Then, we used an image registration‐based approach with the T2‐weighted image as the target to correct for B0 susceptibility induced echo‐planar image distortions. All corrections were performed in the native space of the diffusion‐weighted images. For consistency, all images were reoriented into a common space defined by the T2‐weighted image and appropriate rotations were applied to the b‐matrix. All deformations were computed and applied in a single step to avoid multiple interpolations of the data. Finally, we obtained the fractional anisotropy (FA) and the three eigenvalue maps by fitting a non‐linear diffusion tensor model to the corrected data using the Robust Estimation of Tensors by Outlier Rejection (RESTORE) algorithm. Resulting FA images were processed using tract‐based spatial statistics^39^ including registration of individual FA volumes to the FMRIB58 template and creation of a mean FA‐image that in a next step was thinned to represent the mean FA skeleton thresholded to FA ≥ 0.20.

FA is the most widely used measure of WM microstructure. However, it is impacted by a range of biological factors such as myelination, fiber density, axonal size, and fiber coherence. Therefore, we also projected maps of axial diffusivity (AD) and radial diffusivity (RD) onto the skeleton. Radial diffusivity is a measure of restricted diffusivity across the axonal walls describing the permeability of axonal membranes and therefore serves as an indirect estimate of the axonal myelination level.^40^ Axial diffusivity, which reflects unobstructed diffusivity along the axonal axis, is more indicative of axonal organization.^41^

Hypothesis 1 Alterations in WM microstructure a trait marker of BD.

To test our primary hypothesis of abnormal white matter microstructure in both BD and REL, we used the Permutation Analysis of Linear Models (PALM)^42^ testing for group differences in FA, RD, and AD with age and medication load as nuisance variables. Since the location of the aberrant WM microstructure in BD and REL is a matter of debate, we chose a whole brain approach. For voxel‐wise analyses, 5000 permutations were used. Results were considered significant if they passed a threshold of *P* < .05 using threshold‐free cluster enhancement, a family‐wise error rate correction for multiple comparisons across voxels, and correction over contrasts. Of note, this approach renders an omnibus‐test obsolete. Further, we accounted for the fact that 10 participants were related (2 BD‐REL, 3 REL‐REL) using multi‐level exchangeability blocks. We also used permutation‐based nonparametric combination (NPC) with Fisher's combination of *P*‐values for the joint analysis of the three diffusion metrics in an explorative analysis.^43^ Results of this exploratory analysis are presented in the Supporting Information, S2. For each cluster that passed the significance threshold, the coordinates and *P*‐values of the peak‐voxel (*P*_min_) and the cluster sizes are reported. Results were back‐projected into native FA spaces ensuring that voxels fell within WM pathways for each person.

Hypothesis 2 Differential age effects on WM microstructure.

To determine whether age regression slopes differ between groups, we extracted FA values from three ROIs implicated by previous studies (ie, the genu of the CC, the left and right UNC^21^, ^22^). The ROIs were derived by calculating the overlap between the respective structure taken from John Hopkins University WM atlases (ICBM‐DTI‐81 WM labels atlas for the genu of the CC, and JHU WM tractography atlas thresholded at 25% for UNC) and the mean FA skeleton. Further, we extracted FA from the peak voxels that emerged during group comparison in the context of hypothesis one (body of the CC, left and right CST). Hierarchical linear regression analyses were performed on FA values in these regions using five predictors (two dummy variables coding the three study groups, age, and two dummy variables coding group‐by‐age interaction). Regression diagnostics were performed to test for collinearity, normality, outliers, and leverage. All results were considered significant with *P* < .0083 thereby applying Bonferroni correction for 6 multiple comparisons (genu and body of the CC, left and right UNC, and left and right CST).

Hypothesis 3 WM microstructure predicts difficulties in emotion regulation.

To address the hypothesis that WM microstructure is associated with difficulties in emotion regulation or symptoms reflecting such difficulties, we conducted four linear regression analyses using the Statistical Package for the Social Sciences version 24 (SPSS). We used the total score of the DERS as the criterion. To determine specificity of the findings, three additional models were calculated using the mean of the parent and child ratings of the ARI and SCARED as well as the CGAS score (in the subsample of BD youth and REL) as criteria. Predictors included sex, age, IQ, primary diagnosis (dummy coded as BD: 1 0; REL: 0 1; HV: 0 0), comorbidities (ADHD, anxiety disorders), and FA values extracted from WM tracts previously implicated in BD (genu, body, and splenium of the CC, left and right anterior cingulum bundle, UNC, ATR, SLF, ILF, IFOF, and CST). Regression diagnostics were performed to test for collinearity, normality, outliers, and leverage. All results were considered significant with *P* < .0125 thereby applying Bonferroni correction for four regression models (DERS, ARI, SCARED, CGAS).

### 2.5 Statistical analysis of demographic and questionnaire data

All statistical analyses of clinical data were performed using SPSS. For demographic data conforming to the assumptions of parametric analysis, we used either analysis of variance or Student's *t* test, for nominal demographic data Chi‐square tests were computed. Results were considered significant with *P* < .05.

## 3 RESULTS

Hypothesis 1 Alterations in WM microstructure a trait marker of BD.

**Figure 1:**
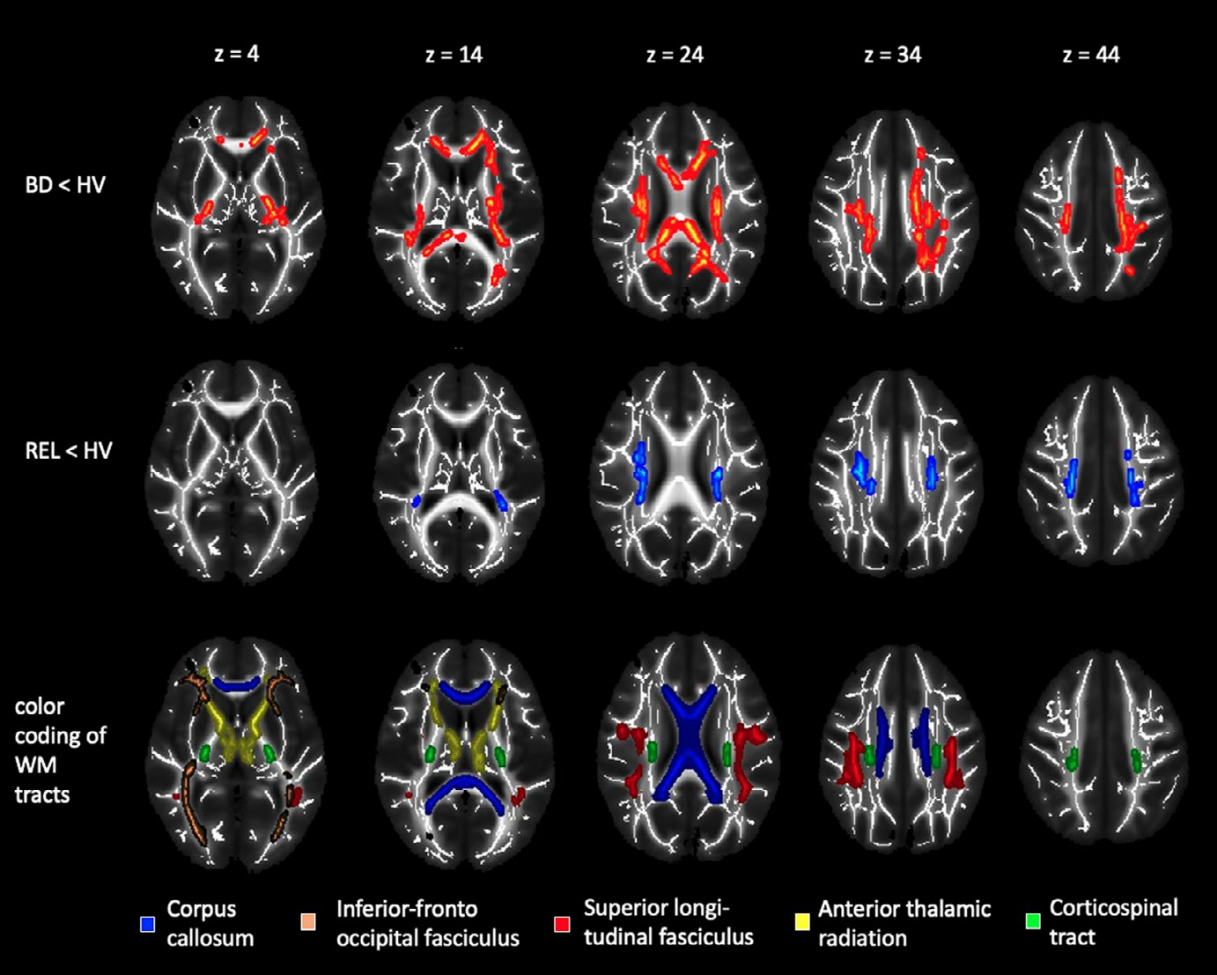
Visualization of group comparisons. Mean maps of fractional anisotropy (greyscale) and mean fractional anisotropy skeletons (white). Red colored voxels signify decreased fractional anisotropy in patients with bipolar disorder compared to healthy volunteers. Blue colored voxels signify decreased fractional anisotropy in relatives of patients with bipolar disorder compared to healthy volunteers. We used the TBSS‐fill script to improve visualization of the results. Images are shown in radiological convention, that is, the left side of the brain is depicted on the right. Maps are thresholded at *P* < .05 FWE‐corrected across voxels and correction over contrasts. The lowest panel shows the corpus callosum taken from the ICBM‐DTI‐81 WM labels atlas and the inferior‐fronto‐occipital fasciculus, superior longitudinal fasciculus, anterior thalamic radiation and corticospinal tract from the JHU WM tractography atlas thresholded at 25% overlaid on the mean FA map of this study to help with the interpretation of the significant cluster. FA, fractional anisotropy; WM, white matter

Compared to HV, individuals with BD showed significantly reduced FA in a large cluster with peak voxels in the left CST (*x* = −24, *y* = −20, *z* = 35, 10 102 voxel, *P*_min_ = .016). This cluster extended to other major tracts such as the CC, ATR, IFOF, UNC, and SLF (Figure 1). Further, we observed reduced FA in REL compared to HV in the CST bilaterally (right: *x* = 25, *y* = −18, *z* = 36, 1119 voxel, *P*_min_ = .02; left: *x* = −25, *y* = −20, *z* = 33, 766 voxel, *P*_min_ = .03) and the body of the CC (*x* = −17, *y* = −3, *z* = 38, 33 voxel, *P*_min_ = .048, Figure 1). Direct comparisons of FA between BD patients and REL yielded no significant results.

Post‐hoc tests on the mean FA values extracted from the significant cluster (BD vs HV), showed no differences between BD youth with and without ADHD (*t*_(34)_ = 0.9, *P* = .37), an anxiety disorder (*t*_(34)_ = 0.6, *P* = .58) or youth with BD‐1 compared to BD‐2 disorder (*t*_(34)_ = 0.4, *P* = .35). In REL, FA reductions in the CST were more pronounced in individuals with a diagnosis of ADHD (FA right CST: *t*_(34)_ = 2.2, *P* = .03; FA left CST: *t*_(34)_ = 2.1, *P* = .04), but the subgroup of REL without any diagnosis also showed significantly lower FA than HV in the right (*t*_(55)_ = −3.56, *P* = .001) and left CST (*t*_(55)_ = −3.75, *P* = 4.2 × 10^−4^). REL with a diagnosis of anxiety did not differ from REL without an anxiety diagnosis in any of the significant clusters (right CST: *t*_(34)_ = 0.86 *P* = .40; left CST: *t*_(34)_ = −0.22, *P* = .83; body of the CC: *t*_(34)_ = −0.72, *P* = .48).

There were no significant group differences with regard to AD and RD. However, we observed marginally higher RD in BD youth compared to HV for the cluster in the CST (*x* = −24, *y* = −20, *z* = 35, 2256 voxel, *P*_min_ = .058).

Hypothesis 2 Differential age effects on WM microstructure.

**Figure 2:**
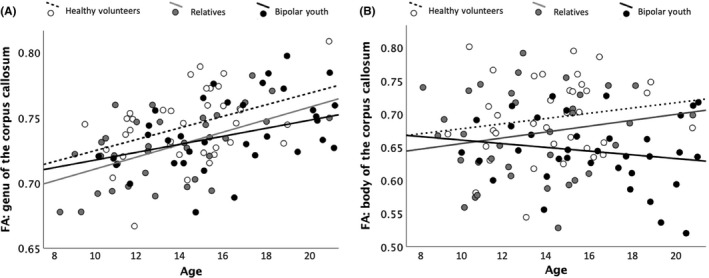
Differential age effect in bipolar youth compared to relatives and healthy volunteers (HVs). A, Shows the association between fractional anisotropy in the genu of the corpus callosum and age for the three study groups separately. B, Illustrates the relationship between fractional anisotropy in the body of the corpus callosum and age for the cluster in the body of the corpus callosum where significant group differences between relatives and HVs were observed

A significant age*group interaction effect was observed in the genu and the body of the CC, indicating steeper age‐related FA increases in HV and REL compared to BD (see Figure 2). There was no significant age*group interaction in the left and right uncinate fasciculus or left and right corticospinal tract.

In more detail, FA in the genu of the corpus callosum was predicted by the dummy variable coding a differential age effect in BD youth (β_stand_ = −0.33, *t* = −3.54, *P* = .001, adjusted *R* ^2^ = .106, *F*_(1,106)_ = 12.52, *P* = .001). FA in the body of the corpus callosum was predicted by the dummy variable coding a differential age effect in BD youth (β_stand_ = −1.13, *t* = −2.89, *P* = .005) and the dummy variable coding the absence of HV status (β_stand_ = −0.75, *t* = −1.92, *P* = .058, adjusted *R* ^2^ = .185, *F*_(2,105)_ = 12.07, *P* = 1.9 × 10^−5^). Age was the sole predictor of FA in the left uncinate fasciculus (β_stand_ = 0.29, *t* = 3.17, *P* = .002, adjusted *R* ^2^ = .086, *F*_(2,105)_ = 10.03, *P* = .002). FA in the left corticospinal tract was predicted by age (β_stand_ = 0.44, *t* = 5.48, *P* = 2.9 × 10^−7^), being related to an individual with BD (β_stand_ = −0.63, *t* = −7.11, *P* = 2.9 × 10^−10^), and diagnosis of BD (β_stand_ = −0.43, *t* = −4.90, *P* = 4.0 × 10^−6^, adjusted *R* ^2^ = .415, *F*_(3,104)_ = 24.63, *P* = 4.0 × 10^−12^). FA in the left corticospinal tract was predicted by being related to an individual with BD (β_stand_ = −0.50, *t* = −5.13, *P* = 1.0 × 10^−6^), and diagnosis of BD (β_stand_ = −0.54, *t* = −5.62, *P* = 1.6 × 10^−7^, adjusted *R* ^2^ = .272, *F*_(2,105)_ = 19.58, *P* = 5.9 × 10^−8^). Exploratory whole brain analyses did not identify additional regions with different age regression slopes.

Hypothesis 3 WM microstructure as predictor of emotional difficulties.

In a first step we analyzed the associations between the four criteria of the regression models (DERS, ARI, SCARED, and CGAS). We observed a positive correlation between the DERS and ARI score (*r* = .62, *P* < .001) as well as the SCARED score (*r* = .44, *P* < .001). Further, the overall DERS score was negatively associated with the CGAS (*r* = −.52, *P* < .001).

**Figure 3:**
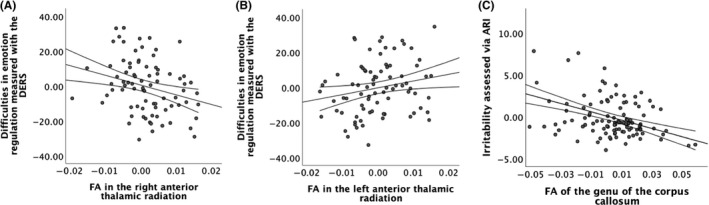
Partial regression plots of fractional anisotropy in (A) the right and (B) the left anterior thalamic radiation and difficulties in emotion regulation as dependent variable and (C) fractional anisotropy in the genu of the corpus callosum and irritability across the entire sample

DERS scores (mean of parent and child ratings) were best explained by diagnosis of BD (β_stand_ = 0.78, *t* = 8.04, *P* = 7.2 × 10^−12^), being related to an individual with BD (β_stand_ = 0.36, *t* = 3.87, *P* = 2.2 × 10^−4^) and diagnosis of an anxiety disorder (β_stand_ = 0.21, *t* = 2.67, *P* = .009; adjusted *R* ^2^ = .549, *F*_(3,82)_ = 34.27, *P* = 2.7 × 10^−14^). Age, sex, IQ, and diagnosis of ADHD did not predict difficulties in emotion regulation (all *P* > .31). In the second step mean FA values of 17 WM tracts were added to the model and FA in the right (β_stand_ = −0.36, *t* = −2.84, *P* = .006) and left ATR (β_stand_ = 0.27, *t* = 2.18, *P* = .032) were retained as significant predictors of DERS‐scores (adjusted *R* ^2^ = .582, *F*_(5,82)_ = 23.79, *P* = 2.2 × 10^−14^; Figure 3; for details see Supporting Information, S3).

Irritability as measured by the ARI (mean of parent and child ratings) was best accounted for by the diagnosis of BD (β_stand_ = 0.43, *t* = 4.54, *P* = 1.5 × 10^−5^) and younger age (β_stand_ = −0.24, *t* = −2.56, *P* = .012; adjusted *R* ^2^ = .158, *F*_(2,105)_ = 10.95, *P* = 4.8 × 10^−5^). Sex, IQ, diagnosis of ADHD, diagnosis of an anxiety disorder or being related to an individual with BD did not predict levels of irritability (all *P* > .08). In a second step, FA values were added to the model. FA in the genu of the CC (β_stand_ = −0.44, *t* = −5.33, *P* = 5.9 × 10^−7^) was added as an additional predictor of irritability (adjusted *R* ^2^ = .333, *F*_(3,104)_ = 18.68, *P* = 9.5 × 10^−10^; Figure 3; for details see Supporting Information, S4).

Anxiety as measured by the SCARED (mean of parent and child ratings) was best accounted for by the presence of an anxiety disorder (β_stand_ = 0.42, *t* = 4.59, *P* = 1.3 × 10^−5^), the diagnosis of bipolar disorder (β_stand_ = 0.30, *t* = 2.59, *P* = .011), being related to an individual with BD (β_stand_ = 0.32, *t* = 3.16, *P* = .002), the absence of ADHD (β_stand model_ = −0.25, *t* = −2.57, *P* = .012), and younger age (β_stand model_ = −0.21, *t* = −2.40, *P* = .018). The overall model explained 29.4% of the variance in anxiety (*F*_(5,101)_ = 9.85, *P* = 1.1 × 10*^−^* ^7^). Indices of WM microstructure did not account for variance in the SCARED (all *P*‐values > .09; for details see Supporting Information, S5). Of note, WM microstructure also did not predict the general level of impairment as measured by the CGAS (*F*_(17,53)_ = 0.62, *P* = .86). Thus, it appears that aberrant WM microstructure in the genu of the CC and bilateral ATR uniquely contributes to difficulties in emotion regulation and irritability, but not anxiety or general impairment.

## 4 DISCUSSION

Compared to HV, BD youth and REL displayed reduced FA in the bilateral CST and the body of the CC and were overall comparable regarding their WM microstructure. Further, BD youth and REL showed more difficulties in emotion regulation and were more irritable than HVs. While the microstructure of the ATR was associated with difficulties in emotion regulation, irritability was related to FA in the anterior CC. As expected, BD youth showed more widespread reductions in FA compared to HV. Further, BD youth displayed less age‐related increase in the FA of the genu and the body of the CC compared to HV and REL.

Hypothesis 1 Alterations in WM microstructure a trait marker of BD.

There were no differences in the WM microstructure between individuals with and at risk for BD. However, compared to HV, both individuals with and at risk for BD showed reduced FA in the CST and CC. In REL, but not BD youth, the findings in the CST were more pronounced in the subgroup with a diagnosis of ADHD, which suggests that abnormalities in this tract might not be specific to BD. This interpretation is consistent with the literature that comprises reports of reduced FA in the CST in individuals with^14^ or at risk^15^ for BD and in individuals with ADHD.^44^ The CST is a major motor pathway, and its microstructure has been positively associated with processing speed.^45^ Thus, altered WM microstructure in the CST might contribute to both reduced processing speed^46^ in BD.

Reduced FA in the body of the CC could not be attributed to other clinical variables, which supports the idea that abnormal WM microstructure in the CC might be a trait marker of BD. Indeed, reduced FA in the CC has emerged as the most robust finding in several meta‐analyses in individuals with BD^11^, ^12^ and has also been reported repeatedly in unaffected REL during late adolescence^15^ and adulthood.^16^ The cluster we identified in REL was located in the body of the CC known to interconnect parietal and temporal cortices. The body of the CC develops into the corona radiata, which has been associated with processing speed and working memory performance.^47^ In individuals with BD, reduced FA was observed in a large cluster spanning the entire CC. Future studies should test whether aberrant microstructure in the CC relates to the reduced processing speed and impaired working memory performance previously reported in REL.^48^

Unlike many studies in adult individuals with BD,^13^ we were unable to attribute reductions in FA to increases in RD, which serves as an indirect estimate of axonal myelination. Thus, abnormalities in RD that are often seen in adult BD might reflect altered myelin plasticity, referring to changes in myelination in response to experiences,^20^ rather than altered developmental myelination. However, more studies are needed to prove this hypothesis.

Hypothesis 2 Differential age effects on WM microstructure.

Consistent with our hypothesis, we observed the age regression slope was reduced for FA in anterior CC in BD only.^21^ Further, comparable age regression slopes between REL and HV is consistent with the literature.^23^ Although longitudinal studies are needed to determine the precise time course of CC maturation in BD, numerous reports of reduced FA in the anterior CC in adults with BD suggest that this is not just a developmental delay in CC maturation, but that reduced FA in the anterior CC continues to represent a disease marker in later life.

Of note, we were unable to replicate findings of differential age effects in the CST^21^ and UNC^21^, ^22^ previously reported in BD youth. It might also be that such effects are only detectable in late adolescence/young adulthood,^22^ particularly in tracts such as the UNC, where age‐related FA increases do not plateau until the 3rd decade of life.^19^ Further, we might have been unable to detect these effects using a cross‐sectional as opposed to a longitudinal design and a TBSS‐ as opposed to a tractographic approach, which might be more sensitive to developmental effects.^21^, ^22^

Hypothesis 3 WM microstructure predicts emotional difficulties.

In contrast to our hypothesis, FA in the ATR, but not in the UNC, was associated with difficulties in emotion regulation, which appear to not be specific to BD or the risk for BD as they were also associated with the diagnosis of anxiety disorders. The ATR is a major projection from the thalamus that carries reciprocal connections from the striatum to the frontal cortex.^49^ The FA in the right ATR has been previously associated with reduced learning from unexpected negative feedback (negative prediction error) and increased risk‐taking.^17^ With regard to emotion regulation, data suggest that during cognitive emotion regulation (ie, distraction, reappraisal) the PFC is able to reduce the impact of the hippocampus, amygdala, and ventral tegmental area on ventral striatal negative prediction error signals, allowing for flexible adaptation of behavior.^50^ Changes in the microstructure of the ATR connecting the PFC with the striatum might impair this flexibility.

Surprisingly, difficulties regulating negative emotions were also associated with increased FA in the left ATR. The left hemisphere has been primarily associated with the approach of positive stimuli, which will likely elicit positive emotions, while the right hemisphere is more involved in inhibiting actions that may lead to negative emotions.^51^ As episodes of elated and depressed mood characterize BD, one might speculate that this lateralization might play a role in the switches between mood states. However, this is an unexpected finding that warrants replication. Additionally, future studies should explore in more detail how structural aberrations in primarily motivational networks affect emotion regulation.

Consistent with a previous study,^24^ FA in the genu of the CC was a significant predictor of irritability. Consistent with previous studies, we observed elevated levels of irritability in BD youth^52^ and to a lesser extent in REL. As outlined above, the anterior CC has been associated with working memory performance and sustained attention.^53^ However, the hypothesis that impaired executive functions might mediate associations between aberrant WM microstructure in the anterior CC and irritability should be investigated in future studies.

### 4.1 Limitations

We did not apply a cardiac‐gated acquisition protocol. Thus, an impact of pulsatile motion artifacts on our results cannot be excluded, although the use of robust tensor fitting renders this unlikely. In addition, most patients were taking psychotropic medication and reported comorbid ADHD or/and anxiety disorders. Although additional analyses suggest that our results in these BD youth are not due to these confounding factors, small effects might have been undetected. Further, we included REL with diagnoses of anxiety and ADHD so as to not recruit an unusually resilient sample. This allowed to test, whether REL with and without diagnoses of ADHD and anxiety are comparable. Finally, a tractographic approach might have been more sensitive in the detecting differential age effects as TBSS projects the maximal value on the WM skeleton and might therefore fail to capture subtle structural abnormalities.

## 5 CONCLUSION

Our results show that aberrations in the microstructure of the CST and the body of the CC, both related to cognitive functions and aberrant emotion processing, are present not only in BD youth but also in relatives of individuals with BD suggesting that they might constitute a trait marker of BD. We also observed lower age‐related FA increases in the anterior CC in BD youth only. Notably, FA in the anterior CC was negatively associated with levels of irritability, whereas FA in the ATR was associated with difficulties in emotion regulation, which we observed in BD youth and REL.

## CONFLICT OF INTERESTS

The author has no conflicts of interest.

## AUTHOR CONTRIBUTION

All authors have made substantial contributions to conception and design, or acquisition of data, or analysis and interpretation of data; and been involved in drafting the manuscript or revising it critically for important intellectual content; and given final approval of the version to be published. Each author agrees to be accountable for all aspects of the work in ensuring that questions related to the accuracy or integrity of any part of the work are appropriately investigated and resolved.

## Data Availability

The data that support the findings of this study are available from the corresponding author upon reasonable request.

## References

[1] American Psychiatric Association . Diagnostic and Statistical Manual of Mental Disorders, 5th edn Arlington, VA: American Psychiatric Publishing; 2013.

[2] Merikangas KR , Akiskal HS , Angst J , et al. Lifetime and 12‐month prevalence of bipolar spectrum disorder in the national comorbidity survey replication. Arch Gen Psychiatry. 2007;64(5):543‐552. 17485606 10.1001/archpsyc.64.5.543 PMC1931566

[3] Mcguffin P , Rijsdijk F , Andrew M , Sham P , Katz R , Cardno A . The heritability of bipolar affective disorder and the genetic relationship to unipolar depression. Arch Gen Psychiatry. 2003;60(5):497‐502. 12742871 10.1001/archpsyc.60.5.497

[4] Mitchell PB , Loo CK , Gould BM . Diagnosis and monitoring of bipolar disorder in general practice. Med J Aust. 2010;193(S4):S10‐S13. 20712553 10.5694/j.1326-5377.2010.tb03890.x

[5] Stensland MD , Schultz JF , Frytak JR . Depression diagnoses following the identification of bipolar disorder: costly incongruent diagnoses. BMC Psychiatry. 2010;10:39. 20525372 10.1186/1471-244X-10-39 PMC2894758

[6] Wessa M , Kanske P , Linke J . Bipolar disorder: a neural network perspective on a disorder of emotion and motivation. Restor Neurol Neurosci. 2014;32(1):51‐62. 23603441 10.3233/RNN-139007

[7] Kennedy N , Everitt B , Boydell J , Van Os J , Jones PB , Murray RM . Incidence and distribution of first‐episode mania by age: results from a 35‐year study. Psychol Med. 2005;35(6):855‐863. 15997605 10.1017/s0033291704003307

[8] Brotman MA , Skup M , Rich BA , et al. Risk for bipolar disorder is associated with face‐processing deficits across emotions. J Am Acad Child Adolesc Psychiatry. 2008;47(12):1455‐1461. 19034190 10.1097/CHI.0b013e318188832e PMC2693273

[9] Strakowski SM , Adler CM , Almeida J , et al. The functional neuroanatomy of bipolar disorder: a consensus model. Bipolar Disord. 2012;14(4):313‐325. 22631617 10.1111/j.1399-5618.2012.01022.x PMC3874804

[10] Basser PJ , Pierpaoli C . Microstructural and physiological features of tissues elucidated by quantitative‐diffusion‐tensor MRI. J Magn Reson B. 1996;111(3):209‐219. 8661285 10.1006/jmrb.1996.0086

[11] Yang C , Lei L , Hu X , et al. Psychoradiologic abnormalities of white matter in patients with bipolar disorder: diffusion tensor imaging studies using tract‐based spatial statistics. J Psychiatry Neurosci. 2018;43(6):170221. 30565904 10.1503/jpn.170221 PMC6306286

[12] Dong D , Wang Y , Chang X , et al. Shared abnormality of white matter integrity in schizophrenia and bipolar disorder: a comparative voxel‐based meta‐analysis. Schizophr Res. 2017;185:41‐50. 28082140 10.1016/j.schres.2017.01.005

[13] Bellani M , Boschello F , Delvecchio G , et al. DTI and myelin plasticity in bipolar disorder: integrating neuroimaging and neuropathological findings. Front Psychiatry. 2016;7:21. 26973545 10.3389/fpsyt.2016.00021 PMC4771723

[14] Yip SW , Chandler RA , Rogers RD , Mackay CE , Goodwin GM . White matter alterations in antipsychotic‐ and mood stabilizer‐naive individuals with bipolar II/NOS disorder. Neuroimage Clin. 2013;3:271‐278. 24273712 10.1016/j.nicl.2013.08.005 PMC3814955

[15] Sprooten E , Sussmann JE , Clugston A , et al. White matter integrity in individuals at high genetic risk of bipolar disorder. Biol Psychiatry. 2011;70(4):350‐356. 21429475 10.1016/j.biopsych.2011.01.021

[16] Mahapatra A , Khandelwal SK , Sharan P , Garg A , Mishra NK . Diffusion tensor imaging tractography study in bipolar disorder patients compared to first‐degree relatives and healthy controls. Psychiatry Clin Neurosci. 2017;71(10):706‐715. 28419638 10.1111/pcn.12530

[17] Linke J , King AV , Poupon C , Hennerici MG , Gass A , Wessa M . Impaired anatomical connectivity and related executive functions: differentiating vulnerability and disease marker in bipolar disorder. Biol Psychiatry. 2013;74(12):908‐916. 23684382 10.1016/j.biopsych.2013.04.010

[18] Roybal DJ , Barnea‐Goraly N , Kelley R , et al. Widespread white matter tract aberrations in youth with familial risk for bipolar disorder. Psychiatry Res. 2015;232(2):184‐192. 25779034 10.1016/j.pscychresns.2015.02.007 PMC6147249

[19] Lebel C , Beaulieu C . Longitudinal development of human brain wiring continues from childhood into adulthood. J Neurosci. 2011;31(30):10937‐10947. 21795544 10.1523/JNEUROSCI.5302-10.2011 PMC6623097

[20] Zatorre RJ , Fields RD , Johansen‐Berg H . Plasticity in gray and white: neuroimaging changes in brain structure during learning. Nat Neurosci. 2012;15(4):528‐536. 22426254 10.1038/nn.3045 PMC3660656

[21] Cabeen RP , Laidlaw DH , Ruggieri A , Dickstein DP . Preliminary mapping of the structural effects of age in pediatric bipolar disorder with multimodal MR imaging. Psychiatry Res Neuroimaging. 2018;273:54‐62. 29361347 10.1016/j.pscychresns.2017.12.006 PMC5815932

[22] Weathers J , Lippard ETC , Spencer L , Pittman B , Wang F , Blumberg HP . Longitudinal diffusion tensor imaging study of adolescents and young adults with bipolar disorder. J Am Acad Child Adolesc Psychiatry. 2018;57(2):111‐117. 29413143 10.1016/j.jaac.2017.11.014 PMC5806147

[23] Ganzola R , Nickson T , Bastin ME , et al. Longitudinal differences in white matter integrity in youth at high familial risk for bipolar disorder. Bipolar Disord. 2017;19(3):158‐167. 28470928 10.1111/bdi.12489

[24] Linke JO , Adleman NE , Sarlls J , et al. White matter microstructure in pediatric bipolar disorder and disruptive mood dysregulation disorder. J Am Acad Child Adolesc Psychiatry. 2019 10.1016/j.jaac.2019.05.035 PMC9686453 31330239

[25] Van Rheenen TE , Murray G , Rossell SL . Emotion regulation in bipolar disorder: profile and utility in predicting trait mania and depression propensity. Psychiatry Res. 2015;225(3):425‐432. 25537486 10.1016/j.psychres.2014.12.001

[26] Hollingshead AB . Four factor index of social status. Unpublished Manuscript, Yale Univeristy 1975.

[27] Kaufman J , Birmaher B , Brent D , et al. Schedule for affective disorders and schizophrenia for school‐age children‐present and lifetime version (K‐SADS‐PL): initial reliability and validity data. J Am Acad Child Adolesc Psychiatry. 1997;36:980‐988. 9204677 10.1097/00004583-199707000-00021

[28] Wechsler D . Wechsler Abbreviated Scale of Intelligence. New York, NY: The Psychological Corporation: Hartcourt Brace & Company; 1999.

[29] Young RC , Biggs JT , Ziegler VE , Meyer DA . A rating scale for mania: reliability, validity and sensitivity. Br J Psychiatry. 1978;133:429‐435. 728692 10.1192/bjp.133.5.429

[30] Poznanski EO , Cook SC , Carroll BJ . A depression rating scale for children. Pediatrics. 1979;64:442‐450. 492809

[31] Stringaris A , Goodman R , Ferdinando S , et al. The affective reactivity index: a concise irritability scale for clinical and research settings. J Child Psychol Psychiatry. 2012;53(11):1109‐1117. 22574736 10.1111/j.1469-7610.2012.02561.x PMC3484687

[32] Birmaher B , Khetarpal S , Brent D , et al. The screen for child anxiety related emotional disorders (SCARED): scale construction and psychometric characteristics. J Am Acad Child Adolesc Psychiatry. 1997;36(4):545‐553. 9100430 10.1097/00004583-199704000-00018

[33] Shaffer D , Gould MS , Brasic J , et al. A children's global assessment scale (CGAS). Arch Gen Psychiatry. 1983;40(11):1228‐1231. 6639293 10.1001/archpsyc.1983.01790100074010

[34] Gratz KL , Roemer L . Multidimensional Assessment of emotion regulation and dysregulation: development, factor structure, and initial validation of the difficulties in emotion regulation scale. J Psychopathol Behav Assess. 2004;26(1):41‐54.

[35] Neumann A , Van Lier PA , Gratz KL , Koot HM . Multidimensional assessment of emotion regulation difficulties in adolescents using the difficulties in emotion regulation scale. Assessment. 2010;17(1):138‐149. 19915198 10.1177/1073191109349579

[36] Sarlls JE , Shaw PJ , Adleman NE , Roopchansingh V . Straightforward method to improve sensitivity in diffusion imaging studies of subjects who move. Paper Presented At: Proc. Intl. Soc. Mag. Reson. Med, Melbourne; 2012.

[37] Le Bihan D , Poupon C , Amadon A , Lethimonnier F . Artifacts and pitfalls in diffusion MRI. J Magn Reson Imaging. 2006;24(3):478‐488. 16897692 10.1002/jmri.20683

[38] Pierpaoli C , Walker I , Irfanoglu MO , et al. An integrated software package for processing of diffusion MRI data. 18th meeting of the international society for magnetic resonance in medicine. 2010:1597.

[39] Smith SM , Jenkinson M , Johansen‐Berg H , et al. Tract‐based spatial statistics: voxelwise analysis of multi‐subject diffusion data. NeuroImage. 2006;31:1487‐1505. 16624579 10.1016/j.neuroimage.2006.02.024

[40] Song S , Sun S , Ramsbottom M , Chang C , Russell J , Cross A . Dysmyelination revealed through MRI as increased radial (but unchanged axial) diffusion of water. NeuroImage. 2002;17:1429‐1436. 12414282 10.1006/nimg.2002.1267

[41] Sun SW , Liang HF , Trinkaus K , Cross AH , Armstrong RC , Song SK . Noninvasive detection of cuprizone induced axonal damage and demyelination in the mouse corpus callosum. Magn Reson Med. 2006;55(2):302‐308. 16408263 10.1002/mrm.20774

[42] Winkler AM , Ridgway GR , Douaud G , Nichols TE , Smith SM . Faster permutation inference in brain imaging. NeuroImage. 2016;141:502‐516. 27288322 10.1016/j.neuroimage.2016.05.068 PMC5035139

[43] Winkler AM , Webster MA , Vidaurre D , Nichols TE , Smith SM . Multi‐level block permutation. NeuroImage. 2015;123:253‐268. 26074200 10.1016/j.neuroimage.2015.05.092 PMC4644991

[44] Svatkova A , Nestrasil I , Rudser K , Goldenring Fine J , Bledsoe J , Semrud‐Clikeman M . Unique white matter microstructural patterns in ADHD presentations‐a diffusion tensor imaging study. Hum Brain Mapp. 2016;37(9):3323‐3336. 27159198 10.1002/hbm.23243 PMC5663221

[45] Dev SI , Nguyen TT , McKenna BS , et al. Steeper slope of age‐related changes in white matter microstructure and processing speed in bipolar disorder. Am J Geriatr Psychiatry. 2017;25(7):744‐752. 28342644 10.1016/j.jagp.2017.02.014 PMC5479871

[46] Mur M , Portella MJ , Martinez‐Aran A , Pifarre J , Vieta E . Long‐term stability of cognitive impairment in bipolar disorder: a 2‐year follow‐up study of lithium‐treated euthymic bipolar patients. J Clin Psychiatry. 2008;69(5):712‐719. 18435565

[47] Bendlin BB , Canu E , Willette A , et al. Effects of aging and calorie restriction on white matter in rhesus macaques. Neurobiol Aging. 2011;32(12):2319.e1‐2319.e11. 10.1016/j.neurobiolaging.2010.04.008 PMC2939965 20541839

[48] Calafiore D , Rossell SL , Van Rheenen TE . Cognitive abilities in first‐degree relatives of individuals with bipolar disorder. J Affect Disord. 2018;225:147‐152. 28829959 10.1016/j.jad.2017.08.029

[49] Mori S , Zhang J . Principles of diffusion tensor imaging and its applications to basic neuroscience research. Neuron. 2006;51(5):527‐539. 16950152 10.1016/j.neuron.2006.08.012

[50] Mulej Bratec S , Xie X , Wang Y , et al. Cognitive emotion regulation modulates the balance of competing influences on ventral striatal aversive prediction error signals. NeuroImage. 2017;147:650‐657. 28040541 10.1016/j.neuroimage.2016.12.078

[51] Hecht D . The neural basis of optimism and pessimism. Exp Neurobiol. 2013;22(3):173‐199. 24167413 10.5607/en.2013.22.3.173 PMC3807005

[52] Wiggins JL , Brotman MA , Adleman NE , et al. Neural correlates of irritability in disruptive mood dysregulation and bipolar disorders. Am J Psychiatry. 2016;173(7):722‐730. 26892942 10.1176/appi.ajp.2015.15060833 PMC11193882

[53] Yamada S , Takahashi S , Ukai S , et al. Microstructural abnormalities in anterior callosal fibers and their relationship with cognitive function in major depressive disorder and bipolar disorder: a tract‐specific analysis study. J Affect Disord. 2015;174:542‐548. 25556672 10.1016/j.jad.2014.12.022

