# Supplementary material for: White matter microstructure in youth with and at risk for bipolar disorder
Source: Bipolar Disord. 2020 Jan 21;22(2):163–73. doi: 10.1111/bdi.12885 (PMC7155105; doi:10.1111/bdi.12885)
Supplement: Supplementary file 1 [file BDI-22-163-s001.docx]

**Supplementary Material**

**S1 Rigid body registration parameters (translation in mm and rotation in degree) for each diagnostic group**


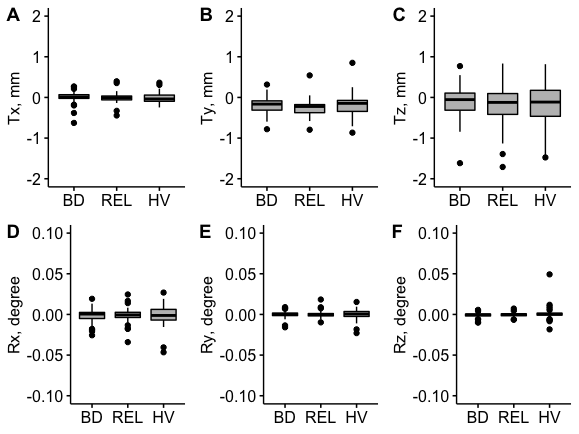


**S2 Results of the exploratory combination of the three diffusion measures**

The exploratory joint analyses of the three diffusion-metrics (FA, AD, RD) showed a more circumscribed pattern of aberrant WM microstructure in BD youth compared to HV. In more detail, the contrast BD youth < HV yielded four clusters in the left CST (x = -24, y = -20, z= 35, 463 voxel, p_min_ = 0.0218), left splenium of the corpus callosum (x = -16, y = -52, z = 26, 355 voxel, p_min_ = 0.0346), left SCR (x = -28, y = -19, z = 21, 63 voxel, p_min_ = 0.0446), and the posterior thalamic radiation (x = -30, y = -40, z = 17, 17 voxel, p_min_ = 0.0478). In the same analyses, we observed four clusters of aberrant WM microstructure in REL. The peaks of these clusters were located in the right CST (x = 21, y = -21, z = 44, 1269 voxel, p_min_ = 0.0068), the left CST (x = -25, y = -19, z = 33, 853 voxel, p_min_ = 0.0154), right SCR (x = -18, y = -5, z = 43, 20 voxel, p_min_ = 0.0478), and right posterior thalamic radiation (x = 36, y = -40, z = 18, 16 voxel, p_min_ = 0.0489).


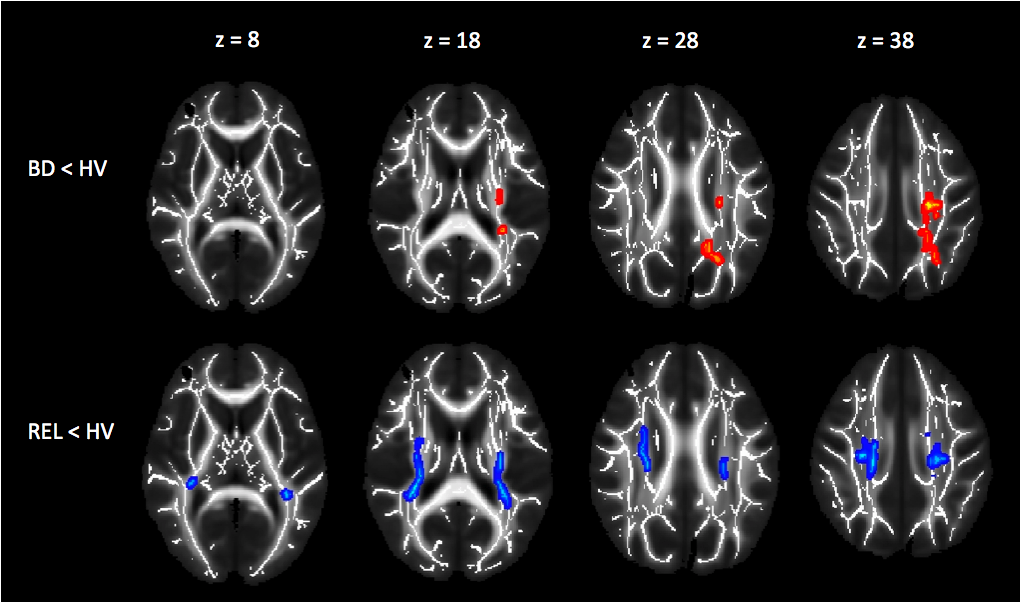


**^Figure S1 Visualization of the exploratory analysis^**^. Mean maps of fractional anisotropy (greyscale) and mean fractional anisotropy skeletons (white).^ ^Red coloured voxels signify aberrant WM microstructure in patients with bipolar disorder compared to healthy volunteers. Blue coloured voxels signify aberrant WM microstructure in relatives of patients with bipolar disorder compared to healthy volunteers. We used the tbss-fill script to improve visualization of the results. Images are shown in radiological convention, i.e., the left side of the brain is depicted on the right. Maps are thresholded at p < 0.05 FWE-corrected across voxels and correction over contrasts.^

**S3 Multiple linear regression analyses predicting difficulties in emotion regulation**

|  | **Coefficients** | | |  | **ANOVA** | |
| --- | --- | --- | --- | --- | --- | --- |
|  | **β** | ***t*** | ***p*** | **Total *R*^2^ Adj** | ***F*** | ***p*** |
| **Model 1: demographics + diagnoses** | | |  |  |  |  |
| Age | -.081 | -1.02 | .312 | 0.547 | 14.96 | < .001 |
| Sex | .060 | 0.79 | .787 |  |  |  |
| IQ | -.051 | -0.64 | .523 |  |  |  |
| BD | .767 | 7.21 | .000 |  |  |  |
| REL | .334 | 3.41 | .001 |  |  |  |
| ADHD | .079 | 0.87 | .390 |  |  |  |
| Anxiety | .190 | 2.19 | .032 |  |  |  |
| **Model 2: significant diagnostic predictors only** | | | | | |  |
| BD | .778 | 7.78 | .000 | 0.549 | 34.27 | < .001 |
| REL | .362 | 3.87 | .000 |  |  |  |
| Anxiety | .214 | 2.67 | .009 |  |  |  |
| **Model 3: significant diagnostic predictors + FA** | | | | | |  |
| BD | .574 | 7.78 | .000 | 0.595 | 7.02 | < .001 |
| REL | .172 | 3.87 | .000 |  |  |  |
| Anxiety | .205 | 2.67 | .009 |  |  |  |
| ATR L | .494 | 2.91 | .005 |  |  |  |
| ATR R | -.450 | -3.21 | .002 |  |  |  |
| CC genu | .032 | 0.37 | .717 |  |  |  |
| CC body | .125 | 1.07 | .291 |  |  |  |
| CC splenium | -.047 | -0.43 | .671 |  |  |  |
| Ant cing bundle L | -.206 | -1.18 | .242 |  |  |  |
| Ant cing bundle R | .084 | 0.55 | .586 |  |  |  |
| IFOF L | -.149 | -0.98 | .331 |  |  |  |
| IFOF R | .178 | 1.19 | .236 |  |  |  |
| ILF L | -.259 | -1.75 | .085 |  |  |  |
| ILF R | .060 | 0.44 | .661 |  |  |  |
| SCR L | -.173 | -1.26 | .212 |  |  |  |
| SCR R | -.132 | -0.92 | .361 |  |  |  |
| SLF L | -.116 | -0.81 | .424 |  |  |  |
| SLF R | .195 | 1.22 | .229 |  |  |  |
| UNC L | -.015 | -0.09 | .930 |  |  |  |
| UNC R | -.123 | -0.74 | .462 |  |  |  |
| **Model 4: significant diagnostic predictors + significant FA predictors** | | | | | |  |
| BD | .711 | 7.40 | .000 | 0.582 | 23.80 | < .001 |
| REL | .302 | 3.26 | .002 |  |  |  |
| Anxiety | .189 | 2.43 | .017 |  |  |  |
| ATR L | .272 | 2.18 | .032 |  |  |  |
| ATR R | -.363 | -2.84 | .006 |  |  |  |

**_Abbreviations: ADHD_**_, Attention-deficit/Hyperactivity disorder;_ **_Adj_**_, adjusted;_ **_ANOVA_**_, analysis of variance;_ **_Ant cing bundle_**_, anterior cingulum bundle;_ **_BD_**_, bipolar disorder;_ **_β_**_, standardized beta coefficient;_ **_CC_**_, corpus callosum;_ **_IFOF_**_, inferior fronto-occipital fasciculus;_ **_ILF_**_, inferior longitudinal fasciculus;_ **_IQ_**_, intelligence quotient;_ **_L_**_, left;_ **_R_**_, right;_ **_REL_**_, relative of an individual with bipolar disorder;_ **_SCR_**_, superior corona radiata;_ **_SLF_**_, superior longitudinal fasciculus;_ **_UNC_**_, uncinate fasciculus_

**S4 Multiple linear regression analyses predicting irritability**

|  | **Coefficients** | | |  | **ANOVA** | |
| --- | --- | --- | --- | --- | --- | --- |
|  | **β** | ***t*** | ***p*** | **Total *R*^2^ Adj** | ***F*** | ***p*** |
| **Model 1: demographics + diagnoses** | | |  |  |  |  |
| Age | -.226 | -2.37 | .020 | 0.195 | 4.57 | < .001 |
| Sex | -.007 | -0.76 | .939 |  |  |  |
| IQ | -.078 | -0.84 | .404 |  |  |  |
| BD | .456 | 3.60 | .001 |  |  |  |
| REL | .179 | 1.59 | .115 |  |  |  |
| ADHD | .001 | 0.01 | .991 |  |  |  |
| Anxiety | .175 | 1.75 | .084 |  |  |  |
| **Model 2: significant diagnostic and demographic predictors only** | | | | | |  |
| Age | -.242 | -2.56 | .012 | 0.158 | 10.95 | < .001 |
| BD | . 429 | 4.54 | .000 |  |  |  |
| **Model 3: significant diagnostic and demographic predictors + FA** | | | | | |  |
| Age | -.225 | -2.16 | .033 | 0.394 | 4.28 | < .001 |
| BD | .326 | 3.52 | .000 |  |  |  |
| ATR L | .136 | 0.72 | .475 |  |  |  |
| ATR R | -.044 | -0.29 | .771 |  |  |  |
| CC genu | -.398 | -4.25 | .000 |  |  |  |
| CC body | -.215 | -1.87 | .066 |  |  |  |
| CC splenium | -.105 | -0.89 | .378 |  |  |  |
| Ant cing bundle L | .079 | -0.46 | .644 |  |  |  |
| Ant cing bundle R | .107 | 0.70 | .489 |  |  |  |
| IFOF L | -.032 | -0.19 | .851 |  |  |  |
| IFOF R | .086 | 0.50 | .619 |  |  |  |
| ILF L | -.258 | -1.67 | .100 |  |  |  |
| ILF R | -.001 | -0.01 | .992 |  |  |  |
| SCR L | -.201 | -1.41 | .162 |  |  |  |
| SCR R | .039 | 0.26 | .797 |  |  |  |
| SLF L | .101 | 0.69 | .492 |  |  |  |
| SLF R | .100 | 0.63 | .532 |  |  |  |
| UNC L | -.235 | -1.41 | .162 |  |  |  |
| UNC R | .017 | 0.10 | .917 |  |  |  |
| **Model 4: significant diagnostic, demographic and FA predictors** | | | | | |  |
| Age | -.225 | -2.16 | .033 | 0.333 | 18.68 | < .001 |
| BD | .312 | 3.59 | .001 |  |  |  |
| CC genu | -.444 | -5.33 | .000 |  |  |  |

**_Abbreviations: ADHD_**_, Attention-deficit/Hyperactivity disorder;_ **_Adj_**_, adjusted;_ **_ANOVA_**_, analysis of variance;_ **_Ant cing bundle_**_, anterior cingulum bundle;_ **_BD_**_, bipolar disorder;_ **_β_**_, standardized beta coefficient;_ **_CC_**_, corpus callosum;_ **_IFOF_**_, inferior fronto-occipital fasciculus;_ **_ILF_**_, inferior longitudinal fasciculus;_ **_IQ_**_, intelligence quotient;_ **_L_**_, left;_ **_R_**_, right;_ **_REL_**_, relative of an individual with bipolar disorder;_ **_SCR_**_, superior corona radiata;_ **_SLF_**_, superior longitudinal fasciculus;_ **_UNC_**_, uncinate fasciculus_

**S5 Multiple linear regression analyses predicting anxiety**

|  | **Coefficients** | | |  | **ANOVA** | |
| --- | --- | --- | --- | --- | --- | --- |
|  | **β** | ***t*** | ***p*** | **Total *R*^2^ Adj** | ***F*** | ***p*** |
| **Model 1: demographics + diagnoses** | | |  |  |  |  |
| Age | -.223 | -2.52 | .013 | 0.304 | 7.43 | < .001 |
| Sex | .141 | 1.67 | .098 |  |  |  |
| IQ | -.072 | -0.84 | .404 |  |  |  |
| BD | .323 | 2.76 | .007 |  |  |  |
| REL | .337 | 3.24 | .002 |  |  |  |
| ADHD | .401 | -2.49 | .015 |  |  |  |
| Anxiety | -.246 | 4.30 | .000 |  |  |  |
| **Model 2: significant diagnostic and demographic predictors only** | | | | | |  |
| Age | -.209 | -2.40 | .018 | 0.294 | 9.85 | < .001 |
| BD | . 297 | 2.59 | .011 |  |  |  |
| REL | .317 | 3.16 | .002 |  |  |  |
| ADHD | -.245 | -2.57 | .012 |  |  |  |
| Anxiety | .422 | 4.59 | .000 |  |  |  |
| **Model 3: significant diagnostic and demographic predictors + FA** | | | | | |  |
| Age | -.130 | -1.20 | .235 | 0.340 | 3.48 | < .001 |
| BD | .222 | 1.72 | .090 |  |  |  |
| REL | .283 | 2.47 | .016 |  |  |  |
| ADHD | -.243 | -2.44 | .017 |  |  |  |
| Anxiety | .449 | 4.63 | .000 |  |  |  |
| ATR L | -.245 | -1.34 | .183 |  |  |  |
| ATR R | .202 | 1.29 | .202 |  |  |  |
| CC genu | -.166 | -1.72 | .088 |  |  |  |
| CC body | .144 | 1.13 | .261 |  |  |  |
| CC splenium | .028 | 0.24 | .813 |  |  |  |
| Ant cing bundle L | .216 | 1.20 | .232 |  |  |  |
| Ant cing bundle R | -.264 | -1.65 | .103 |  |  |  |
| IFOF L | -.094 | -0.53 | .591 |  |  |  |
| IFOF R | .297 | 1.67 | .100 |  |  |  |
| ILF L | -.116 | -0.81 | .424 |  |  |  |
| ILF R | -.080 | -0.53 | .599 |  |  |  |
| SCR L | -.090 | -0.59 | .560 |  |  |  |
| SCR R | -.044 | -0.26 | .793 |  |  |  |
| SLF L | .178 | 1.19 | .236 |  |  |  |
| SLF R | .032 | 0.20 | .846 |  |  |  |
| UNC L | .024 | 0.14 | .888 |  |  |  |
| UNC R | .007 | 0.04 | .967 |  |  |  |

**_Abbreviations: ADHD_**_, Attention-deficit/Hyperactivity disorder;_ **_Adj_**_, adjusted;_ **_ANOVA_**_, analysis of variance;_ **_Ant cing bundle_**_, anterior cingulum bundle;_ **_BD_**_, bipolar disorder;_ **_β_**_, standardized beta coefficient;_ **_CC_**_, corpus callosum;_ **_IFOF_**_, inferior fronto-occipital fasciculus;_ **_ILF_**_, inferior longitudinal fasciculus;_ **_IQ_**_, intelligence quotient;_ **_L_**_, left;_ **_R_**_, right;_ **_REL_**_, relative of an individual with bipolar disorder;_ **_SCR_**_, superior corona radiata;_ **_SLF_**_, superior longitudinal fasciculus;_ **_UNC_**_, uncinate fasciculus_
